# Monitoring atmospheric particulate matters using vertically resolved measurements of a polarization lidar, in-situ recordings and satellite data over Tehran, Iran

**DOI:** 10.1038/s41598-020-76947-w

**Published:** 2020-11-18

**Authors:** Hossein Panahifar, Ruhollah Moradhaseli, Hamid Reza Khalesifard

**Affiliations:** 1 Department of Physics, Institute for Advanced Studies in Basic Sciences, Zanjan, 4513766731 Iran; 2 Physics Department, Faculty of Science, Zanjan Branch, Islamic Azad University, Zanjan, Iran; 3 Center for Research in Climate Change and Global warming, Institute for Advanced Studies in Basic Sciences, Zanjan, 4513766731 Iran

**Keywords:** Environmental impact, Atmospheric science

## Abstract

The highly polluted atmosphere above Tehran has been investigated by using a polarization lidar operating at 532 nm, in-situ particulate matter suites distributed over the city, and meteorological observations. The measurement campaign is conducted from Nov. 2014 to Jan. 2016. Three typical cases are studied in detail where, the atmosphere is polluted with urban pollution, mixture of urban pollution and dust particles from local sources, and long range transported dust from the Arabian Peninsula. For these cases, vertical profiles of the lidar backscatter coefficient, extinction coefficients, particle depolarization ratio ($\delta _{\text {p}}$) and mass concentrations of atmospheric aerosols (separated into dust and non-dust particles) are presented. Using the lidar recordings, variations of the planetary boundary layer height above the city are investigated along the year. During November to February, lidar profiles frequently show polluted boundary layers that are reaching up to 1 km above the ground level. The depolarization ratio ($\delta _{\text {p}}$) varies between 0.04 and 0.08 in the polluted boundary layer. During the campaign, for 103 days the urban pollution was dominant, 45 recorded dust events ($0.15<\delta _{\text {p}}<0.20$) were originated from the dry regions in the south of Tehran and 15 dust events ($0.20<\delta _{\text {p}}<0.35$) impacted the city that were originated from the Arabian Desert and Mesopotamia.

## Introduction

Tehran ($\sim 35.55^{\circ }$–$35.83^{\circ }$ N, $\sim 51.09^{\circ }$–$51.59^{\circ }$ E) is the capital and the most populated city in Iran. More than 13.2 million inhabitants are living in the Tehran metropolitan area^1^. Air pollution is a major environmental issue for the city and particulate matter (PM) concentrations frequently exceed healthy levels based on the world health organization (WHO) standards^2^. Intense urbanization, industrialization, and increase of fossil fuel usage lead to almost permanent aerosol loading in the whole atmosphere of the city^3,4^. This high aerosol load from local sources beside meteorological factors such as stable atmosphere and temperature inversion leads to severe air pollution in Tehran^5^.

To investigate urban pollution in Tehran, some researches have been done by use of emission models, synoptic meteorological, and in-situ PM concentration measurements. Arhami et al. collected daily ${{\text {PM}}_{2.5}}$ samples at main stations in Tehran from Feb. 2014 until Feb. 2015. They characterized chemical compositions of the samples and assess the major source categories in Tehran. Their research showed that dominant components of the ${{\text {PM}}_{2.5}}$ in weight are: organic matters (35%), dust (25%), non-sea salt sulfates (11%), elemental carbon (9%), ammonium (5%) and nitrate (2%). They also identified 5 major sources responsible for 81% of the fine PM variations including industrial sources, combustion sources, residual oil combustion, vehicles, and soil dust^6^. Shahbazi et al. developed a traffic-related emission inventory of Tehran using a combination of traffic and emission rates modeling. Their results indicate that driving cars are main sources for emission of carbon monoxide (CO), volatile organic compounds (VOC), nitrogen oxides (NOx), and sulfur oxides (SOx)^7^. Furthermore, Zawar-Reza et al. used of spatio-temporal emission pattern of particulate matters recorded by in-situ PM monitoring stations and concluded that vehicles’ traffic and meteorological conditions are responsible for escalations in particulate matter concentrations^8^. The city of Tehran also lies in the middle of the global dust belt^9,10^, so in addition to urban pollution, it is highly probable to be affected by dust storms originated from either local or trans-regional sources especially during spring and summer^11–14^. To identify sources and transport paths of mineral dust masses to Tehran’s atmosphere, some studies have been conducted by use of in-situ and synoptic meteorological data, satellite data analysis, aerosol tracking models, and 3-D numerical modeling of aerosols dispersion^15–17^. All above-mentioned studies revealed valuable information about the particle types and concentrations at ground level, but still do not provide any information about vertical profile of the atmospheric particulate matters.

To provide insight into vertical distribution of atmospheric aerosols, lidar measurements are quite powerful^18–22^. Li et al. studied planetary-boundary-layer-height (PBLH) variation using automated lidar and ceilometers (ALC) in the New York city and explored the correlation with ${{\text {PM}}_{2.5}}$ measured at ground level. They found a strong inverse relation between the PBLH and ${{\text {PM}}_{2.5}}$ concentration during the morning transition period in the New York city^21^. Filioglou et al. conducted lidar measurements in the United Arab Emirates and reported lidar ratio of $42\pm 5$ sr and depolarization ratio of $31\pm 2$ % at 532 nm for the Arabian dust^19^. Hofer et al. performed comprehensive lidar measurements in Dushanbe, Tajikistan. They reported lidar ratio of $36\pm 2$ sr and depolarization ratio of $31\pm 2$ % at 532 nm for the Arabian dust transported to Central Asia^20^. Heese et al. used the ${{\text {Polly}}^{XT}}$ lidar to investigate highly polluted atmosphere over the Pearl River Delta, Guangzhou, China^22^. For urban pollution aerosol, they reported lidar ratios of 30 to 80 sr with a mean value of $48.0\pm 10.7$ sr at 532nm. In that report, the linear particle depolarization ratio at 532 nm for urban pollution lies mostly below 5%, with a mean value of $3.6\pm 3.7\%$. Burton et al. also used recordings of a high spectral resolution lidar equipped with polarization channels, and classified different atmospheric aerosols based on their intensive parameters including the lidar ratio, depolarization ratio and backscatter color ratio^23^.

For the first time, in this work we are reporting results of thirteen months of lidar measurements on atmospheric aerosols in Tehran. The research includes vertically resolved polarization lidar measurements supported by space-borne remote sensing data as well as synoptic meteorological and in-situ PM concentration measurements. The polarization lidar technique permits the discrimination of desert dust from urban pollution and other aerosol types^22–26^. This research provides valuable information about the aerosol vertical distribution, boundary layer height variations, aerosol typing based on the particle linear depolarization ratio ($\delta _{\text {p}}$) of lidar signal, and source apportionment of particulate matter in the atmosphere above Tehran. So that a clear view regarding natural and anthropogenic aerosol contributions to the overall aerosol conditions is possible. This paper includes three sections while “Observation site and methodology” is dedicated to explanation of the observation site, methodologies of the measurements and data-set. Lidar measurements and obtained results are discussed in “Results and discussions”, and the paper ends with a summary and conclusion in “Summary and conclusion”.

## Observation site and methodology

Climatology of Tehran, ground-based facilities, calculation techniques, satellite data and atmospheric models that have been used in this study are described in the following.

### Tehran climatology

**Figure 1:**
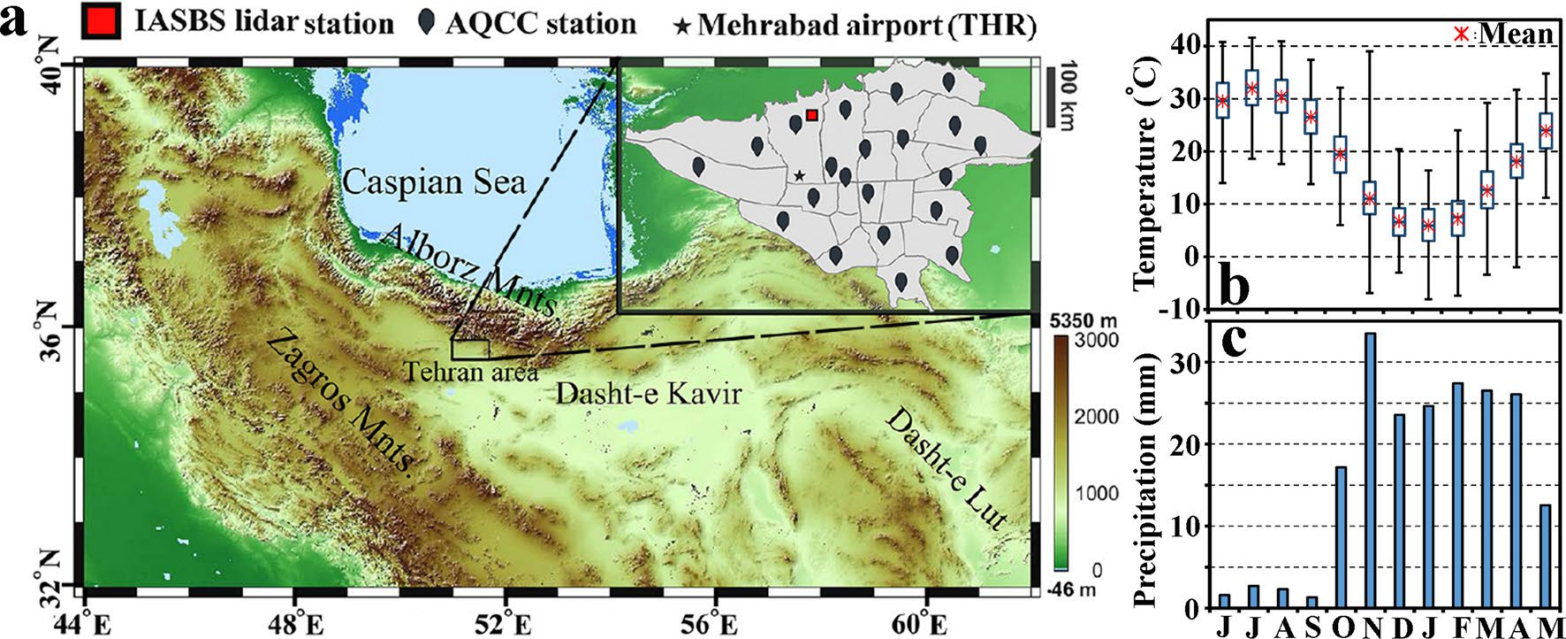
(**a**) Orography of the northern part of Iran provided by Shuttle Radar Topography Mission project (SRTM), the location of lidar (red square), the Mehrabad synoptic station (black asterisk, WMO code: OIII) and the AQCC stations (black balloons) in municipal district of Tehran. (**b**) Boxplot of temperature ($^{\circ }$C), lower and upper box boundaries 25th and 75th percentile respectively, line inside box indicates median, lower and upper lines shows maximum and minimum respectively and red stars show the mean of data. (**c**) Monthly average of precipitation (mm) at Mehrabad stations between 2010 and 2018.

Tehran is located in the northern part of the Iran Plateau ($\sim 26^{\circ }$–$35^{\circ }$ N, $\sim 46^{\circ }$–$61^{\circ }$ E) and on the foothills of Alborz mountains. Figure 1a shows the regional orography and geographical location of Tehran, as well as locations of measurement sites including IASBS lidar station (red square), Mehrabad synoptic station (black star) and Air Quality Controlling Company (AQCC) stations (black balloons). Figure 1a illustrates that the greater Tehran metropolitan is more expanded toward the west and south. The region also is faced to Alborz mountains in its north side. The existence of such a high altitude barrier affects the wind and dispersion pattern of pollutants over Tehran. The elevation across the city ranges from 900 m above mean sea level (amsl) in the south to 1800 m amsl in the northern part. Such a variation of elevation across the city makes the meteorological conditions quite different at its various points. Tehran’s complex topography is one of the factors which aggravates the city air pollution problem.

Variations of monthly averages of surface temperature and precipitation in Tehran are shown in Fig. 1b,c. These data are recorded at the Mehrabad meteorological station (WMO code: OIII) during the years 2010-2018. According to theses results, Tehran has a semi-arid climate^27,28^. Figure 1b illustrates that temperature increases up to $43\;^{\circ }$C in July and drop to as low as $-6.5\;^{\circ }$C in January. The minimal rainfall occurs from June to September, and the highest value of monthly averaged precipitation occurs in November (Fig. 1c).

Demographically, Tehran has an estimated 8.7 million inhabitants and the larger Tehran metropolitan with 16 sub-provinces has a population estimated at 13.2 million^1^. Although roughly 11% of Iran’s total population lives in the city of Tehran, they consume more than 20 million liters of fuel per day which is 22% of total fuel consumption in Iran. According to reports by the statistical center of Iran, there are around 4 million cars, 3 million motorcycles, and around 20 million daily trips in the city^1^.

### Ground-based measurements

An elastic backscatter polarization lidar operating at 532 nm is used as the main ground-base remote sensing instrument in this research. The lidar station is located at $35.7669^{\circ }$N, $51.3118^{\circ }$E, at an altitude of 1430 m amsl in the northwest of Tehran and inside the urban area (Fig. 1a). The measurement campaign lasted from Nov. 2014 until Jan. 2016. The lidar was working continuously in 24/7 operation mode except during precipitation conditions, electricity failures and repair of the lidar components. There are total of 357 days of lidar measurements during campaign. Technical specifications of the lidar are summarized in Table 1. The lidar is constructed in a bi-axial structure and the transmitter–receiver complete overlap starts at 150 m above the ground level (agl). The lidar transmitter repetition rate is 2 Hz and its data are captured with vertical resolution 12 m and stored with a temporal resolution 7 min. Based on the specifications of the optical and electronic elements that have been used in the detection unit of the lidar, the cross talk between parallel and perpendicular channels is less than 1.0%.

**Table 1:** Technical specification of the portable polarization lidar.

**Transmitter**		
Laser	Type	Frequency doubled pulsed Nd:YAG
	Model	Quantel, Ultra CFR
	Energy per pulse at 1064nm/532nm (mJ)	50/30
	Repetition Rate (Hz)	1–20
	Divergence (mrad)	$<6$
	Pulse duration (ns)	10
Beam expander	Type	Galilean Geometry
	Model	Thorlabs, LC2632
	Beam expansion ratio	10×
	Divergence of expanded laser beam (mrad)	$< 1$
**Receiver**		
Telescope	Type	Modified Cassegrian Geometry
	Model	VIXEN, VMC200L
	Primary mirror diameter (mm)	200
	Effective Focal length (mm)	1950
Photomultiplier	Parallel channel	ElectronTubes 9789QB
	Perpendicualr channel	Electrontubes 9658B
	Cross talk between parallel and perpendicular receiving channels (%)	$<1$
**Signal acquisition system**		
Oscilloscope	Type	Digital oscilloscope
	Model	Tektronix, TDS3054
	Maximum count rate (MHz)	350
	Time bin width (ns)	20

The Mehrabad meteorological station (OIII) is located at the distance of 8 km toward the south of the lidar station and mostly has the same distance to the central district of Tehran. Regular synoptic and upper air measurements are carrying at the Mehrabad station. Radiosonde balloons are lunching twice a day at 00:00 and 12:00 UTC at this station and measuring altitude, vertical profile of pressure, temperature, relative humidity (RH) and wind (both wind speed and wind direction) at specific pressure levels. In surface observation section, the horizontal visibility (HV), temperature, wind speed and direction, the RH and precipitation amounts are measured every 3 h and are used in this study.

Tehran Air Quality Control Company (AQCC) data are also used as another ground-based in-situ measurement. During the measurement campaign, the AQCC was using of 21 automated air monitoring stations and measuring concentration of main pollutant species almost continuously. The location of each AQCC station are shown in Fig. 1a. From these measurements, concentrations of carbon oxides (CO and ${{\text {CO}}_{2}}$), Nitrogen oxides (NO and ${{\text {NO}}_{2}}$) and particulate matter smaller than 10 and 2.5 micrometer (${{\text PM}_{10}}$ and ${{\text PM}_{2.5}}$) can be obtained. In this study we just used of the ${{\text PM}_{10}}$ and ${{\text PM}_{2.5}}$ recordings. The accuracy of these recordings are $1\;{\upmu} {{\text{g/m}}^{3}}$ as reported by the AQCC. All the concentration levels mentioned in this study shows their corresponding hourly averaged values over all AQCC active stations in Tehran.

### Retrieval of particle optical properties using lidar measurements

A height-resolved separation of dust and non-dust particles is possible by using recordings on the parallel and perpendicular channels of a polarized-elastic lidar at 532 nm. The methodology is applicable to cases where only two basic types of aerosols exist. It is usually assumed that the atmospheric aerosols are a mixture of dust and non-dust particles. These aerosols should be known in terms of their corresponding depolarization and lidar ratios^29–31^. As the first step, the total particle backscatter coefficient $(\beta _{\text {p}})$ that is a sum of backscatter coefficients at parallel and perpendicular channels can be retrieved by applying the Klett–Fernald method^32^. Then, as it is shown by Freudenthaler et al., the particle depolarization ratio $(\delta _{p})$ is computed using the measured volume depolarization ratio $(\delta _{\text {v}})$ and the derived particle backscatter coefficient $(\beta _{\text {p}})$^33^. Afterward using the procedure proposed by Tesche et al., the profile of the total particle backscatter coefficient can be decomposed to dust and non-dust components ($\beta _{\text {d}}$ and $\beta _{\text {nd}}$) through the Eqs. 1a–c^26^:

1a $$\beta _{{\text{d}}} = \beta _{{\text{p}}} \frac{{(\delta _{{\text{p}}} - \delta _{{{\text{nd}}}} )(1 + \delta _{{\text{d}}} )}}{{(\delta _{{\text{d}}} - \delta _{{{\text{nd}}}} )(1 + \delta _{{\text{p}}} )}}{\text{ for }}\delta _{{{\text{nd}}}} < \delta _{{\text{p}}} < \delta _{{\text{d}}} ,{\text{ and }}\beta _{{{\text{nd}}}} = \beta _{{\text{p}}} - \beta _{{\text{d}}}$$

1b $$\beta _{{\text{d}}} = \beta _{{\text{p}}} {\text{ for }}\delta _{{\text{p}}} \ge \delta _{{\text{d}}}$$

1c $$\beta _{{{\text{nd}}}} = \beta _{{\text{p}}} {\text{ for }}\delta _{{\text{p}}} \le \delta _{{{\text{nd}}}}$$

To solve Eq. 1a, the dust depolarization ratio $(\delta _{\text {d}})$ and the non-dust depolarization ratio $(\delta _{\text {nd}})$ should be presumed as 0.31 and 0.05 respectively^29–31^. In the final step, as shown by Mamouri et al., the mass concentrations of dust ($M_{\text {d}}$) and non-dust ($M_{\text {nd}}$) particles can be obtained by applying appropriate values for particle density ($\rho$), extinction-to-volume conversion factors ($c_{\text {v},\lambda }$) and lidar ratio (S) through the Eq. 2^31,34^:

2a $$M_{{\text{d}}} = \rho _{{\text{d}}} c_{{{\text{v}},d,\lambda }} \beta _{d} {\text{S}}_{d}$$

2b $$\begin{aligned} M_{\text {nd}}=\rho _{\text {nd}} c_{{{\text{v}},{\text{nd}},\uplambda }}\beta _{\text {nd}}S_{\text {nd}} \end{aligned}$$

The parameters applied to convert particle backscatter coefficient of different aerosol types to corresponding mass concentration are listed in Table 2^19,20,22,23,30,31,35–37^.

**Table 2:** Parameters applied to convert particle backscatter coefficient to mass concentration.

Aerosol type	Parameters			References
	Lidar ratio (sr)	$c_{\text {v},\lambda } (10^{-12}\text {Mm})$	$\rho \;({\text{g/cm}}^{3} )$)	
Middle Eastern dust	40	0.79	2.6	Mamouri et al.^54^, Mamouri and Ansmann^31^, Filioglou et al.^19^
Urban Pollution	40–80	0.25	1.55	Hofer et al.^20,36^, Heese et al. ^22^, Burton et al.^23^
Salt (dry and wet)	25	0.65	1.1	Haarig et al.^37^, Mamouri and Ansmann^30^

### Planetary boundary layer height calculation

To have a better understanding of atmospheric pollution episodes in Tehran, the planetary boundary layer height (PBLH) is retrieved from lidar recordings^38,39^. Prior to any processing on range corrected lidar signal on the parallel channel ($S(R)_{\parallel }$), an algorithm is applied to improve the signal-to-noise ratio. This algorithm is based on shark smell optimization method^40^, and is very important to apply it before running the algorithm to find the boundary layer height. Between 150 and 1000 m agl, the sliding average is performed over each 840 lidar signals (7 min of recording). After averaging, the second derivative of $S(R)_{\parallel }$ with respect to the range has been calculated and its minimum is taken as the top of the boundary layer. The relative error on the retrieved PBLH is between 5 and 10% depending on the atmospheric conditions, and higher errors correspond to more clean atmosphere. It should be noted that the cloud layers also may show the same behavior on the second derivative, but usually they are appearing at higher altitudes. The obtained PBLH by this technique is in good agreement with the boundary layer derived from radiosonde ascents via the Richardson method^39^.

### Satellite data and atmospheric models

The regional distribution and transport of dust aerosols are described using following satellite products. The collection 6 of MODIS level—3 daily products with resolution $1^{\circ } \times 1^{\circ }$ are used to monitor the aerosol loading over the study region and influential sources^41^. These products include aerosol optical depth (AOD) at 550 nm (MOD08-MYD08) and deep blue Ängström Exponent for land (412–470 nm). The MCD19A2 data product with resolution $1\times 1$ km, which is a Multi-Angle Implementation of Atmospheric Correction (MAIAC) algorithm-based on Level-2 gridded (L2G) aerosol optical thickness product also has been used in this study^42^. Details of MODIS Deep Blue aerosol optical depth uncertainties are reported by Sayer et al.^43^. CALIOP retrieved aerosol depolarization ratio and aerosol subtype are compared with ground-base lidar measurements^44,45^. In the “Supplement Movie”, the emission and subsequent transport of dust plumes are shown in the RGB dust composite. This products is produced by use of three IR channels of Spinning Enhanced Visible and Infrared Imager (SEVIRI) on Meteosat Second Generation (MSG) payload. Dust appears pink or magenta in this RGB combination. The full disc view includes the whole of Europe, all of Africa and Middle East and allows frequent sampling every 15 min, with a spatial resolution of 3 km in the nadir^46^. The basic atmospheric parameters such as wind speed and direction at different pressure levels which are used in this research produced by the global atmospheric reanalysis model, ERA-Interim, produced by the European Centre for Medium-Range Weather Forecasts (ECMWF)^47,48^. The back-trajectories are calculated with the NOAA Hybrid Single Particle Lagrangian Integrated Trajectory (HYSPLIT) model^49^.

## Results and discussions

The following results and discussions are based on lidar measurements carried out in Tehran from Nov. 2014 to Jan. 2016. To illustrate frequently observed layers in the atmosphere above Tehran, three cases are presented in “Case studies”. The monthly variation of boundary layer height, air quality index, and surface wind speed are presented in “Boundary layer height evolution”. Source apportionment and aerosol type categorization are also discussed in “Source apportionment”.

### Case studies

To describe the variety of observed aerosol layers in Tehran, three illustrative cases are discussed in the following subsections. In these cases, the particle backscatter coefficient and particle linear depolarization ratio are retrieved based on method described in “Retrieval of particle optical properties using lidar measurements”. To separate dust and non-dust backscatter coefficients, as Mamouri et al. assumed, we use values of 0.31 and 0.05 for dust and non-dust depolarization ratio ($\delta _{\text {d}}$ and $\delta _{\text {nd}}$). The mass concentration profiles are also calculated and Table. 2 used in different cases for setting the conversion factors. The first case, 26–29, December 2014, is representing a period in which the atmosphere of Tehran was heavily polluted with anthropogenic aerosols. The second case, 12–15, December 2014 is a situation comprises of a mixture of urban pollution and dust from local sources. The process of emission and long-range transport of dust plumes from the Arabian Peninsula and Mesopotamia to Tehran are discussed as the last case, 22 April 2015.

#### Case 1: 26–29 December 2014

**Figure 2:**
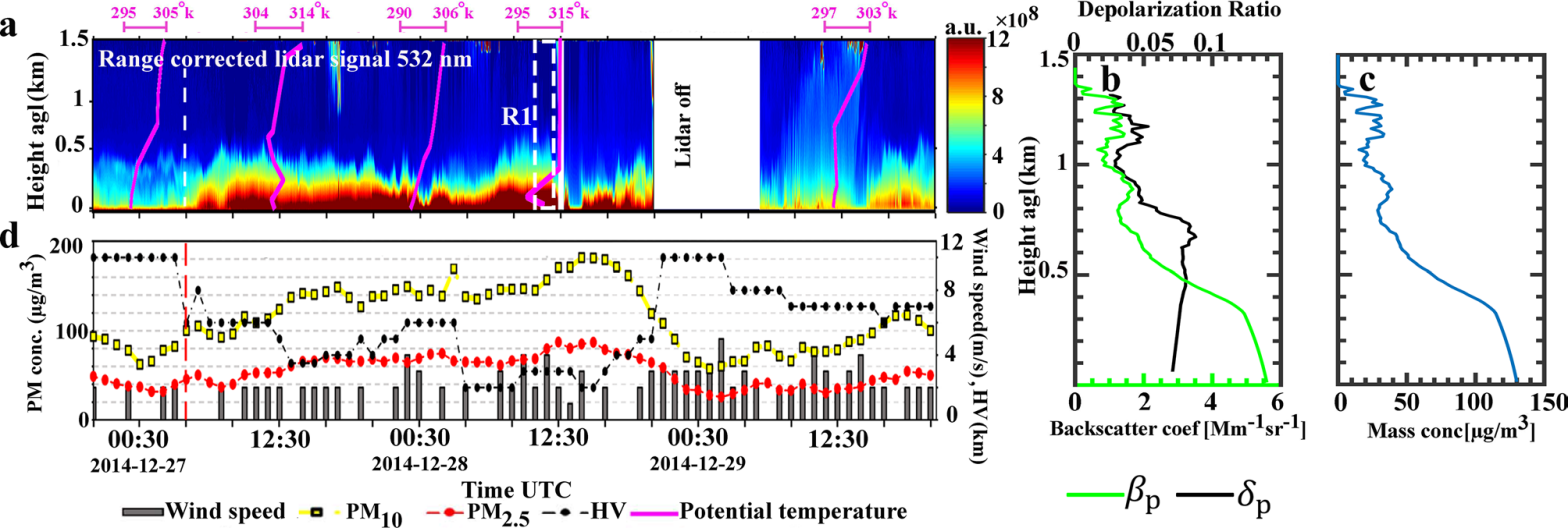
(**a**) Parallel channel time-height series of range corrected lidar signal at 532 nm from 20:30 UTC 26 Dec. to 20:30 UTC 29 Dec. 2014 and potential temperature vertical profiles. (**b**) Derived particle backscatter coefficient (green) and particle depolarization ratio (black) at 532 nm for 11:00 to 12:00 UTC, 28 Dec. 2014 (R1 in Fig. 2a). (**c**) Mass concentration profile obtained from lidar measurement for period same as Fig. 2b with assumed $\rho _{\text {nd}}= 1.55$ ${\text {g}}/{{{\text cm}^{3}}}$, $c_{{{\text{v}},{\text{nd}},\uplambda }} = 0.25 \times 10^{-12}$ Mm and $S_{\text {nd}}=60$ sr. (**d**) Temporal evolution of the horizontal visibility (HV) and surface wind speed recorded at the Mehrabad synoptic station and hourly averages of PM concentrations reported by the AQCC Tehran. The approximate start time of the pollution episode is shown by the white and red dashed lines in (**a**, **d**).

In this case, the lidar was in operation from 26 until 29 Dec. 2014, during the development of an air pollution episode in Tehran. By comparison of lidar and other ground-based recordings, it can be deduced that the pollution episode started at 04:30, 27 Dec. and lasted until 20:30 UTC, 28 Dec. 2014. The approximate time in which the pollution episode starts is shown by the white and red dashed lines in Fig. 2a,d. Air quality guidelines for particulate matter from the WHO stipulates that 24-h mean of ${{\text PM}_{2.5}}$(${{\text PM}_{10}}$) concentrations should not exceed 25(50) ${\upmu} {{\text{g/m}}^{3}}$^2^, while mean of ${{\text PM}_{2.5}}$(${{\text PM}_{10}}$) concentrations are $\sim$ 60(128) ${\upmu} {{\text{g/m}}^{3}}$ during air pollution episode.

The time-height variations of the range corrected lidar signal for the parallel channel at 532 nm are shown in Fig. 2a. The figure spans the time interval of 20:30 UTC, 26 Dec. until 20:30 UTC, 29 Dec. 2014. The vertical profiles of potential temperature are also shown by pink lines in Fig. 2a. It is evident that the potential temperature for most of the time either increases or stays constant with height and this leads to statically stable atmospheres^50^. Figure 2a depicts an atmospheric temperature inversion up to $\sim$700 m agl and even a quite stable atmosphere at higher altitudes at 00:00 UTC on 27 December. The lidar time series (Fig. 2a) clearly shows that after 04:30 UTC on 27 Dec., the strength of lidar signals from near surface layer gradually increases. Vertical profiles of the potential temperatures show that the atmosphere remains stable during the whole pollution episode.

Vertical profiles of optical properties and mass concentration of atmospheric aerosols, for 1 h averaged lidar signal (R1 in Fig. 2a), are shown in Fig. 2b,c. The total particle backscatter coefficient is retrieved by applying the Klett method and shown by the green line in Fig. 2b. Considering that only anthropogenic particles exist in the atmosphere, an initial constant lidar ratio of 60 sr are assumed for these particles. Generally, pollution particles are relatively small, spherical, and highly absorbing that produce low depolarization and large lidar ratios^22,23,25^. The retrieved particle backscatter coefficient profile also shows that the pollution is trapped in a $\sim 500$ m thick boundary layer. This is also in agreement with the potential temperature profile (pink line in R1, Fig. 2a) that shows an inversion layer extended from surface to $\sim$200–500 m agl. The particle linear depolarization ratio at 532 nm (black line in Fig. 2b) is $\sim$0.07 for the polluted boundary layer, which confirms that this aerosol layer is barely depolarizing the lidar signals. The computed particle depolarization ratio ($\delta _{\text {p}}\sim 0.07$) is comparable with non-dust depolarization ratio ($\delta _{\text {nd}}\sim 0.05$). So it can be deduced that the urban pollution is dominant in the atmosphere. Finally, the mass concentration profile is calculated by Eq. 2b where values of $\rho _{\text {nd}}$, $c_{{{\text{v}},{\text{nd}},\uplambda }}$ and $S_{\text {nd}}$ for urban pollution are obtained from Table. 2. The calculated mass concentration is largest ($\sim 130$ $\upmu {\text{g/m}}^{3}$) at near surface and gradually decreases by height. Figure 2d depicts that the ${{\text PM}_{2.5}}$(${{\text PM}_{10}}$) concentrations reaches to $\sim 80(150)$ $\upmu {\text{g/m}}^{3}$ at 12:00 UTC, 28 December 2014. Noting that the PM values are averaged over the city, the mass concentration retrieved from lidar measurements is in a good agreement with in-situ measurements.

As the pollution episode starts, the horizontal visibility at the Mehrabad station drops to $\sim 6$ km and even falls to $\sim 2$ km in the last hours of pollution episode. The surface wind speed is almost less than 3 m/s during the whole episode. After 20:30 UTC, 28 Dec. precipitation starts in the city and the lidar is turned off. The rainfall makes the atmosphere clean and ${{\text PM}_{2.5}}$ (${{\text PM}_{10}}$) drops to below 40(80) $\upmu {\text{g/m}}^{3}$ and the horizontal visibility increases to 10 km (Fig. 2d). This typical case shows how the development of an atmospheric inversion in coincidence with slow surface wind makes the atmosphere quite stable and traps the urban pollution below $\sim$500 m agl (Fig. 2a). The HYSPLIT backward trajectories (not shown here) also confirm that atmospheric aerosols for such cases should be originated from local sources. These kinds of events are occurring very frequently in the late autumn and winter times in the atmosphere above Tehran.

#### Case 2: 12–15 December 2014

**Figure 3:**
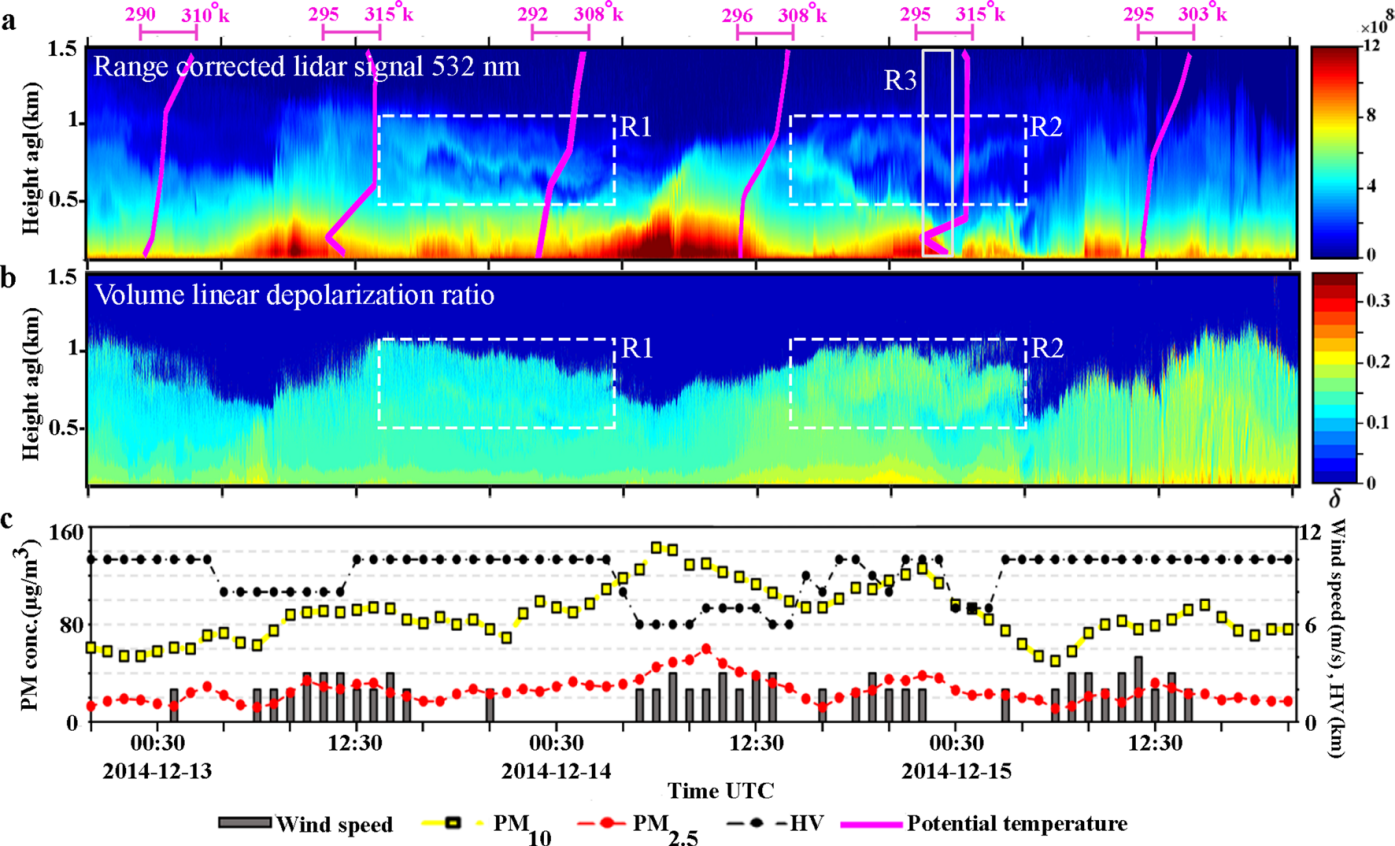
(**a**, **b**) Parallel channel and volume depolarization ratio time-height series of range corrected lidar signal at 532 nm. (**c**) Temporal evolution of the horizontal visibility (HV) and surface wind speed recorded at the Mehrabad synoptic station and hourly averages of PM concentrations reported by the AQCC Tehran, 20:30 UTC, 12 Dec. to 20:30 UTC, 15 Dec. 2014.

Temporal evolution of range corrected lidar signals for the parallel channel, and time-height developments of the volume depolarization ratio for this case are depicted in Fig. 3a,b respectively. Lidar time series are recorded from 20:30 UTC, 12 Dec. to 20:30 UTC, 15 Dec. 2014. Vertical profiles of potential temperature captured at the Mehrabad station are also shown by pink lines in Fig. 3a. The diurnal cycle and the developments of polluted boundary layer on 13 and 14 Dec., are evident on lidar recordings (Fig. 3a). These polluted layers are associated with high emission of anthropogenic aerosols during Tehran rush hours. Vertical profiles of the potential temperature during 13–14 Dec., beside the surface wind speed which blows very slowly (Fig. 3c), confirm that the atmosphere is completely stable. Atmospheric stability leads to an increase in concentrations of particulate matter close to ground level and eventually escalates the strength of backscatter lidar signals. Rectangles R1 and R2 in Fig. 3a include two layers with quite similar behavior. On the other hand, Fig. 3b clearly illustrates that these layers have different volume depolarization ratio. The volume depolarization ratio is $\sim 0.1$ for layer inside R1 and $\sim 0.2$ for layer inside R2.

**Figure 4:**
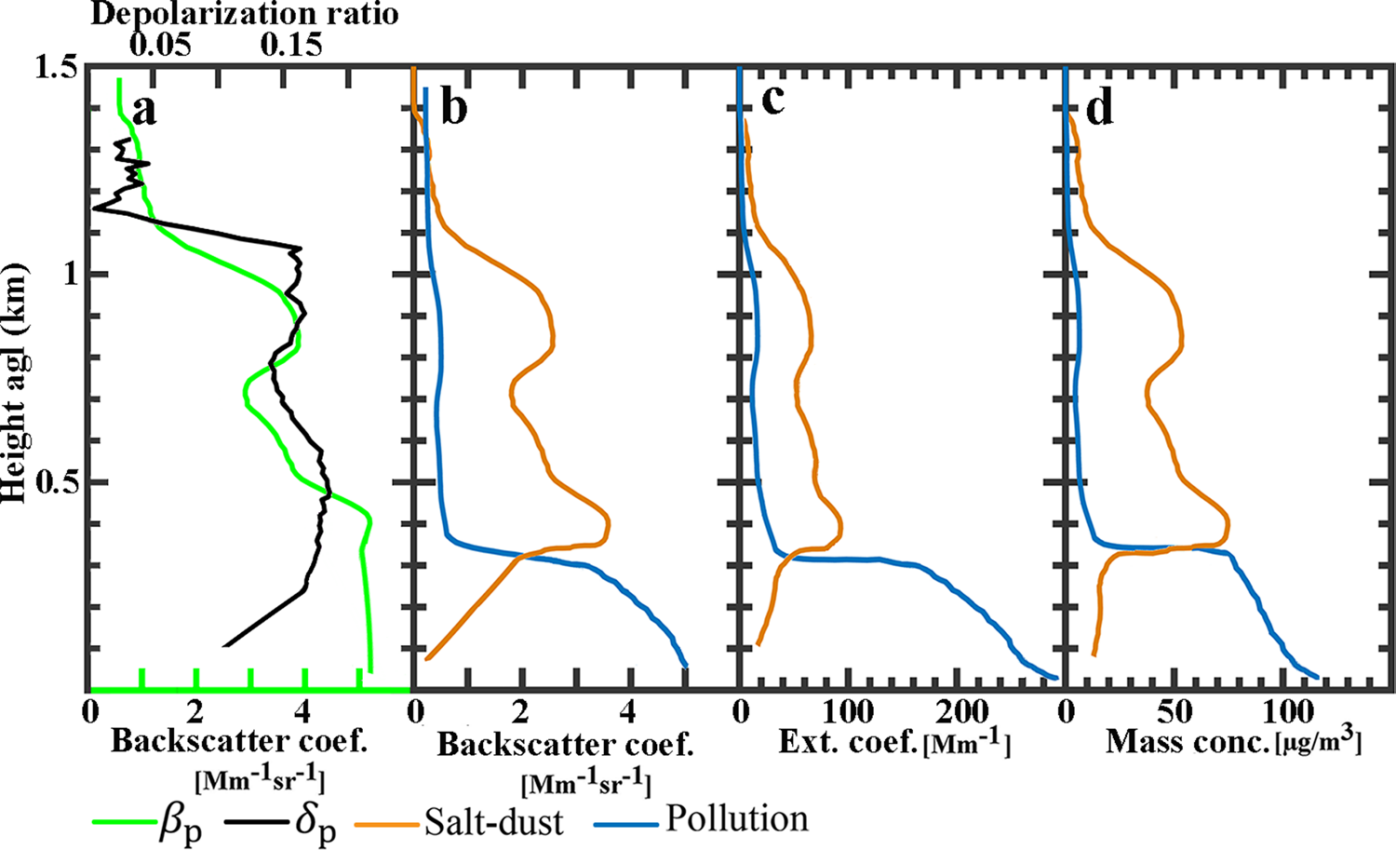
Optical and physical properties of atmospheric aerosols retrieved from averaged lidar signals at 532 nm during 23:00 to 24:00 UTC, 14 Dec. 2014, (R3 in Fig. 3a). (**a**) Particle backscatter coefficient (green) and depolarization ratio (black). (**b**) Backscatter coefficient for salt-dust (orange) and pollution (blue) particles. (**c**) Extinction coefficient for salt-dust and pollution particles by multiplying the backscatter coefficients in Fig. 4b with the lidar ratio of 25 sr for salt-dust and 60 sr for pollution. (**d**) Mass concentration profile obtained from lidar measurement for period same as in Fig. 4b with assumed $\rho _{{{\text{salt}}}} = 1.1\;{\text{g/cm}}^{3}$, $c_{{{\text{v}},{\text{salt}},{\uplambda} }} = 0.65$ Mm for salt-dust and ρ = 1.55 g/cm^3^, $c_{{{\text{v}},{\text{nd}},\uplambda }} = 0.25 \times 10^{-12}$ Mm for pollution.

Some of vertically resolved optical and physical properties of atmospheric aerosols during 23:00 to 24:00 UTC on 14 Dec. 2014 (R3 in Fig. 3a) are shown in Fig. 4. The particle backscatter coefficient (green line in Fig. 4a) is retrieved by applying the Klett method to 1h averaged lidar signals. The backscatter coefficient profile shows a double-layer structure for atmospheric aerosols. The first layer is the polluted boundary layer and extended from surface up to $\sim 500$ m agl. Also a thin aerosol layer is detected at $\sim 900$ m agl. The particle depolarization ratio (black line in Fig. 4a) is also computed and the double-layer structure is clear in its profile. The depolarization ratio is less than $\sim 0.10$ for polluted boundary layer, and it is $\sim 0.17$ for the second layer. Figure 4b shows the backscatter coefficient profiles of the detected aerosol layers after their decomposition into dust and non-dust particles (Eq. 1). The orange line in Fig. 4b shows two layers of dust particles at $\sim 400$ m and $\sim 900$ m agl.

**Figure 5:**
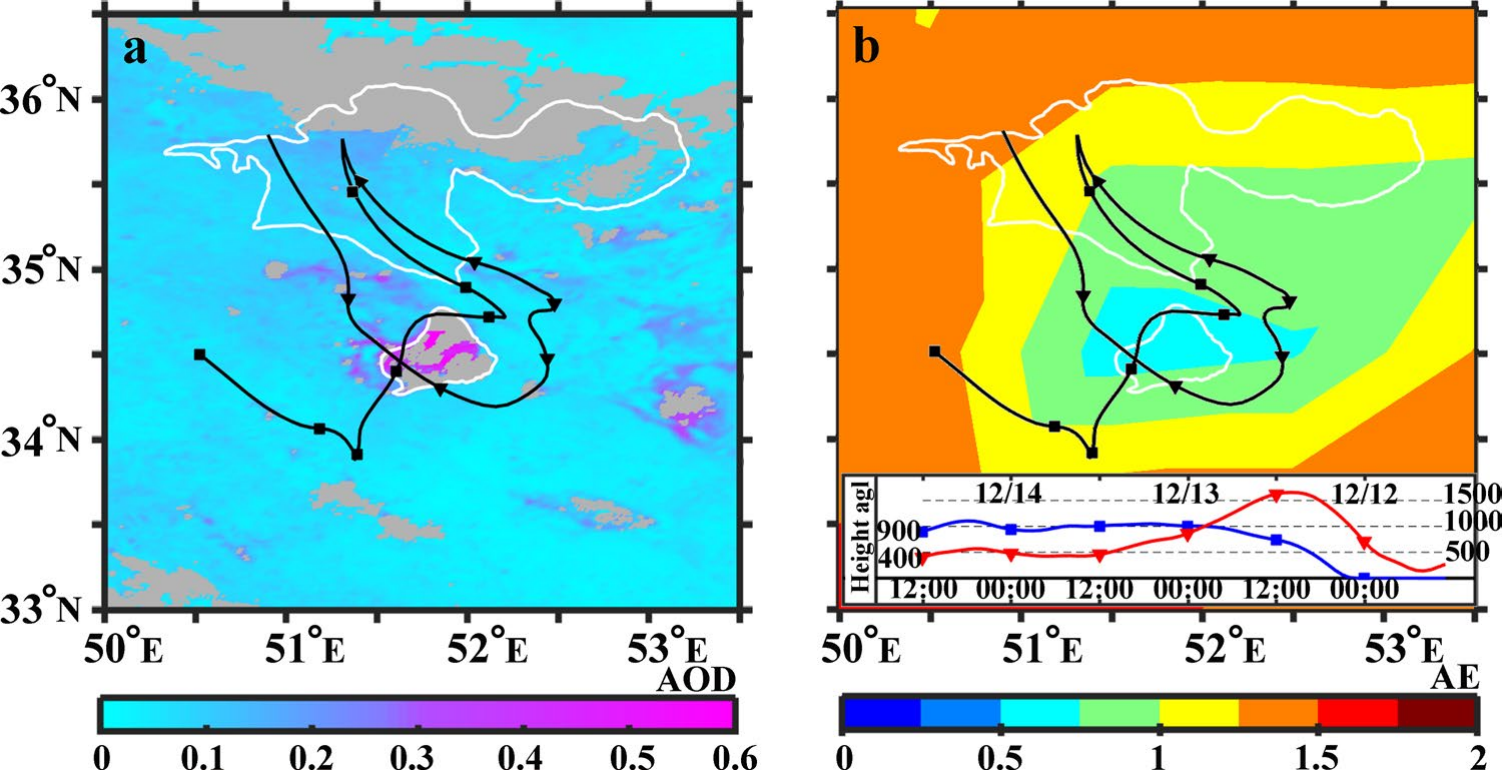
(**a**) 72 hour HYSPLIT backward trajectories arriving at lidar station at 15:00 UTC, 14 Dec 2014 at 900 m and 400 m agl height overlaid on MODIS daily Deep blue AODs with 1 km resolution (MCD19A2 product) on 13 Dec 2014, Grey pattern means no data regions. (**b**) Same back-trajectories as part (**a**) overlaid on Deep blue Ängström exponent on 13 Dec 2014. HYSPLIT heights and dates information are similar for both parts and shown in part (**b**).

The HYSPLIT model and complementary satellite data have been used to determine the source and type of dust particles inside R3. The 72h HYSPLIT backward trajectories started at 15:00 UTC, 14 Dec. 2014, at altitudes of 900 m agl and 400 m agl are shown in Fig. 5. Both trajectories are returning back to deserts in the south of Tehran or northern boarder of Dasht-e-Kavir desert where the dried Qom-Salt-Lake is located. Particles that may rise from the dried bed of such lakes or their coastal area contains considerable amounts of mineral salts and we call such types of particles as salt-dust^51^. Figure 5 shows that the lower trajectory almost rounds the lake and the upper one is crossing over its northern part. On the other hand, Fig. 5a,b illustrate daily MODIS deep blue AODs with 1 km resolution (MCD19A2 product) and deep blue Ängström exponent (AE) with $1^{\circ }$ resolution on 13 Dec. 2014, respectively. These figures depict that the AOD increase ($0.4 \le {\text {AOD}} \le 0.6$) and Ängström Exponent decrease (${\text {AE}}\sim 0.5$) over the dried salt lake on 13 Dec. 2014. The increase (decrease) of the AOD (AE) indicate an increase in the loading of coarse-mode particles over the lake^52^. Referring to Fig. 5, we may conclude that the observed dust layers inside R2 in Fig. 3a are originated from the dry regions in the south of Tehran where a dry salt lake also exists.

Noting to the particle depolarization ratio (black line in Fig. 4a, $\delta _{\text {p}}\sim 0.17$) and the expected source of dust particles, we concluded that these might be some types of salt-dust particles and by referring to the work by Haarig et al., a lidar ratio of $S=25$ sr is considered for them^37^. The respective extinction coefficients for salt-dust and non-dust particles (Fig. 4c) are obtained by multiplying the backscatter coefficient profiles with the lidar ratio of 25 sr for salt-dust and 60 sr for pollution particles. Having in hand all required input parameters of Eq. 2, ($\rho _{{{\text{salt}}}} = 1.1\;{\text{g/cm}}^{3}$, $c_ {\text{v}},{\text{salt}},\uplambda =0.65\times 10^{-12}$ Mm for salt-dust and $\rho _{{{\text{nd}}}} = 1.55\;{\text{g/cm}}^{3}$, $c_{{{\text{v}},{\text{nd}},\uplambda }}=0.25\times 10^{-12}$ Mm for non-dust particles) concentration profiles of salt-dust and non-dust particles are retrieved and depicted in Fig. 4d. The obtained mass concentration of particles at ground level ($M_{\text {nd}}\sim 116 \upmu {\text{g/m}}^{3}$), is in agreement with the obtained averaged ${{\text PM}_{10}}$ concentration value for the same time period in Fig. 3c (${{\text PM}_{10}}\sim 120$ $\upmu {\text{g/m}}^{3}$). As a conclusion, the existence of particles having depolarization ratios $\sim 0.17$ which is close to that of salt particles^37^, suggests that the emission of salt-dust particles from dried Qom-Salt-Lake have an impact on the aerosol contents of the Tehran’s atmosphere. Lidar observations have been conducted by Hofer et al. also reveals that direct emission and transport of resuspended salt dust originated from desiccating lakes have a sensitive impact on the aerosol background optical properties over Dushanbe, Tajikistan^53^.

#### Case 3: 22 April 2015

**Figure 6:**
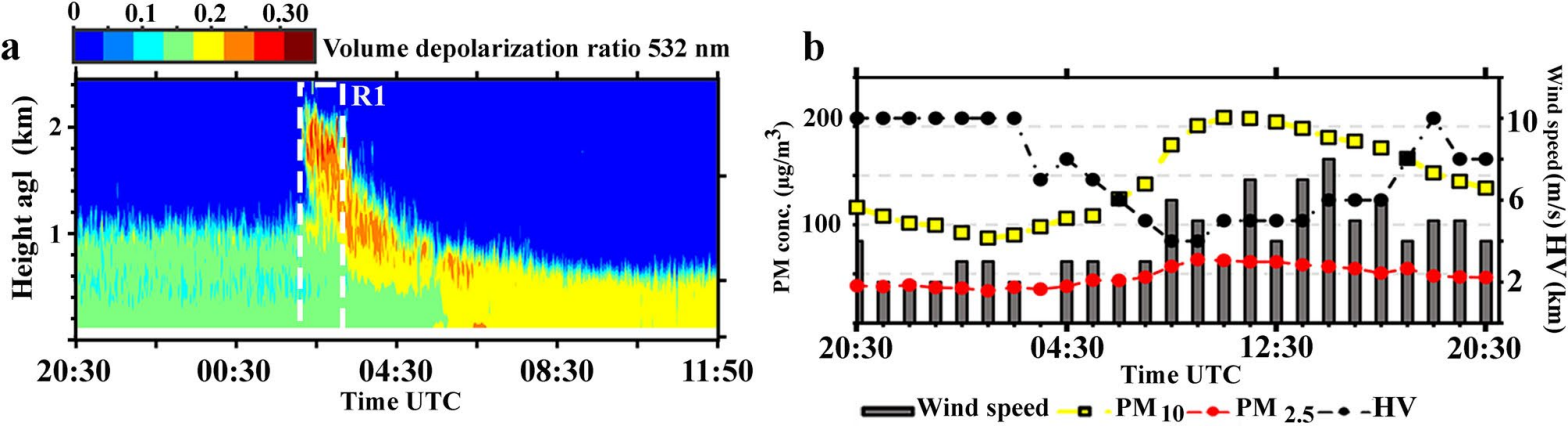
(**a**) Volume depolarization ratio time-height series at 532nm between 20:30 UTC, 21 April until 11:50 UTC on 22 April 2015. (**b**) Temporal evolution of the horizontal visibility (HV) and surface wind speed recorded at the Mehrabad synoptic station and hourly averages of PM concentrations reported by the AQCC Tehran, on 22 April 2015.

An elevated dust layer originated from the Arabian Peninsula and regions in the south of Iraq is transported toward Tehran and arrived to the city on 22 April 2015. The time-height development of the volume depolarization ratio at 532 nm is shown in Fig. 6a. The time-series have been recorded from 20:30 UTC, 21 April to 11:50 UTC, 22 April 2015. This storm caused a power outage in the city and the lidar is turned off at 11:50 UTC. The arrived dust layer is observed at altitudes of 500-2500 m agl around 02:30 UTC on 22 April, and in the following hours, the dust layer is descending toward the surface. Figure 6b illustrates as the dust storm slams the city, the surface wind speed and ${{\text PM}_{10}}$ concentration simultaneously raise to higher than 8 m/s and 200 $\upmu {\text{g/m}}^{3}$ respectively. All these developments cause horizontal visibility drops to lower than 4 km at the Mehrabad station.

**Figure 7:**
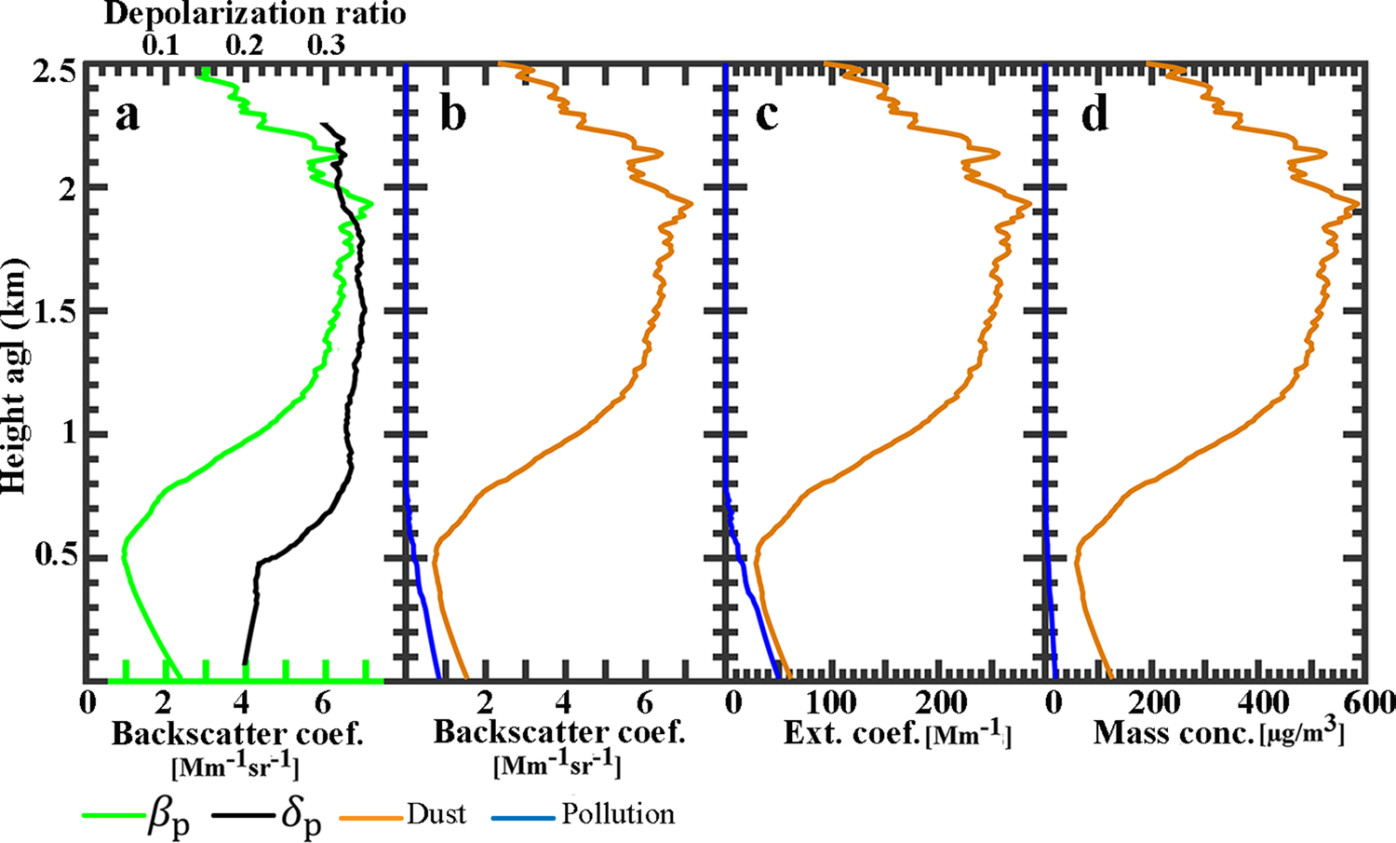
Optical and physical properties of atmospheric aerosols retrieved from averaged lidar signals at 532 nm during 02:00 to 03:00 UTC, 22 April 2014, (R1 in Fig. 6a). (**a**) Particle backscatter coefficient (green) and depolarization ratio (black). (**b**) Backscatter coefficient for dust (orange) and pollution (blue) particles. (**c**) Extinction coefficient for dust and pollution particles by multiplying the backscatter coefficients in Fig. 6b with the lidar ratio of 40 sr for dust and 60 sr for pollution. (**d**) Mass concentration profile obtained from lidar measurement for period same as in (**b**) with assumed ρ_d_ = 2.6 g/cm^3^, $c_ {\text{v}},{\text{d}},{\uplambda}= 0.79\times 10^{-12}$ Mm for dust and ρ_nd_ =1.55 g/cm^3^, $c_{{{\text{v}},{\text{nd}},\uplambda }}=0.25\times 10^{-12}$ Mm for pollution.

The vertical profiles of optical properties and mass concentration are retrieved by performing the approach described in “Retrieval of particle optical properties using lidar measurements” on 1h averaged lidar signals (R1 in Fig. 6a) and its results are shown in Fig. 7. The total particle backscatter coefficient (green line in Fig. 7a) is retrieved using the Klett method by considering lidar ratio 40 sr that is reported for Middle Eastern dust^35,54^. The backscatter coefficient profile shows that the main dust layer extended between 500 and 2500 m agl. The particle depolarization ratio (black line in Fig. 7a) is on average $\sim$0.2 for the first 500 m above the ground, and it is on average $\sim$0.31 for the main dust layer. So it is concluded that the main layer consists of pure dust particles with depolarization ratio of 0.30- 0.35 at 532 nm^35,54^. Knowing that the boundary layer should be polluted with urban pollution, dust and non-dust particles are separated by the technique presented in “Retrieval of particle optical properties using lidar measurements”, and the resolved dust and pollution backscatter coefficient profiles are presented in Fig. 7b. The respective extinction coefficients (Fig. 7c) are obtained by multiplying the backscatter coefficients (in Fig. 7b) with the lidar ratio of 40 sr for dust and 60 sr for pollution particles. Finally, the mass concentration profiles (Fig. 7d) is obtained by applying appropriate values for particle densities and extinction-to-volume conversion factors for dust and urban pollution from Table 2. The mass concentration profile shows that dust mostly contributes to particle mass concentration profile from 500m to 2500 m agl, while non-dust particles extended from surface up to 500 m agl. The dust mass concentration reaches to a maximum of $\sim 600 \upmu {\text{g/m}}^{3}$ at $\sim$1.9 km agl.

**Figure 8:**
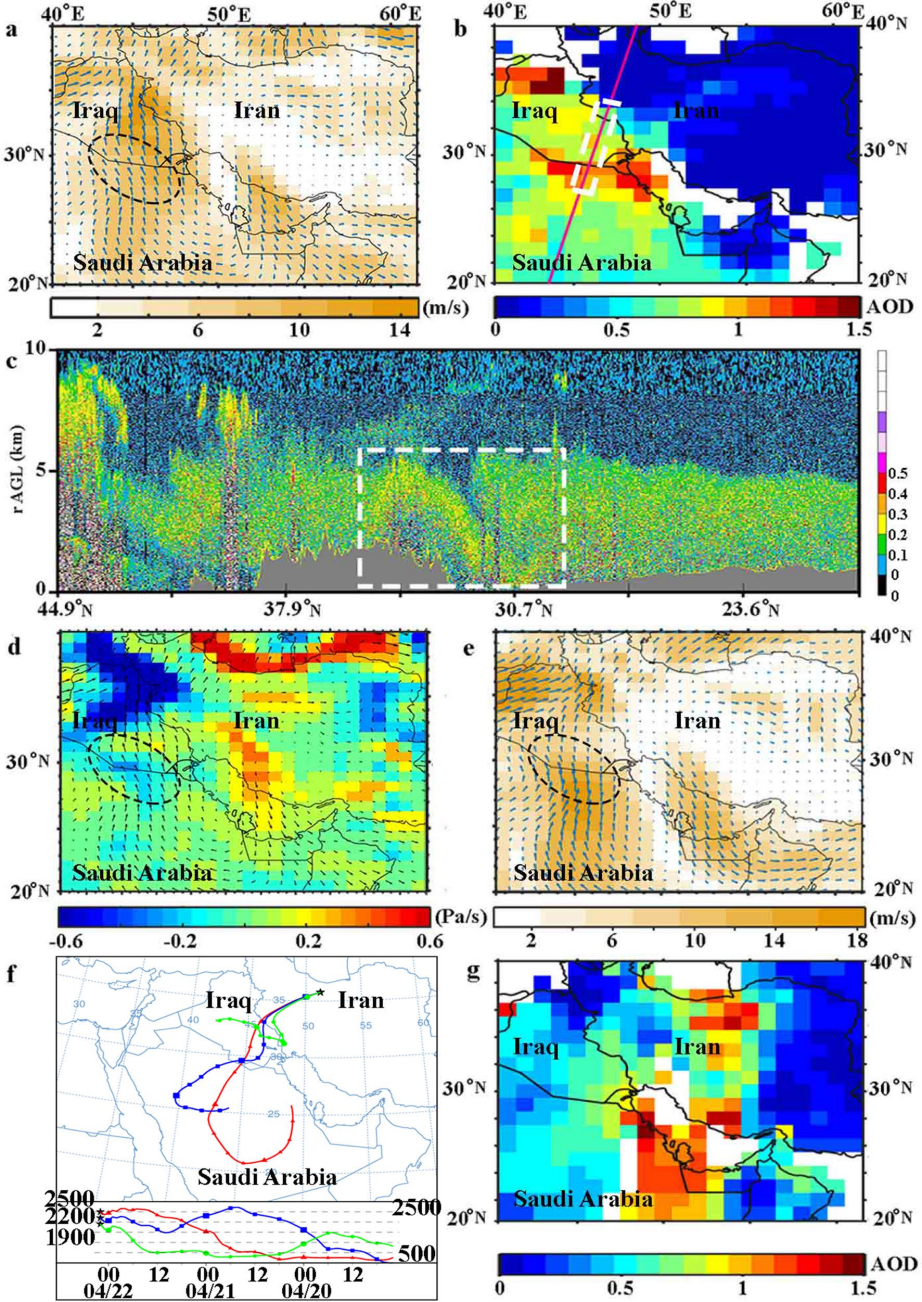
(**a**) ECMWF reanalysis of surface wind speed (color) and direction (arrows) on 06:00 UTC 21 April 2015. (**b**) AQUA MODIS daily deep blue AOD at 550 nm on 21 April 2015 and a daytime CALIPSO ground-track ( solid red line). (**c**) Vertical cross section of CALIOP depolarization ratio at 532 nm on 21 April 2015. (**d**) Same as part (**a**) but at 850  hPa. (**e**) ECMWF atmospheric vertical velocity at 850 hPa (color) and wind direction (arrows) on 06:00 UTC 21 April 2015. (**f**) 72-h HYSPLIT backward trajectories arriving to Tehran at 02:00 UTC on 22 April 2015 at 2500 m, 2200 m and 1900 m heights. (**g**) TERRA MODIS daily deep blue AOD at 550 nm on 22 April 2015.

The emission and subsequent transport of this lofted dust layer can be clearly observed and followed in the RGB composite images from MSG-SEVIRI products (“Supplement Movie”). This movie clearly illustrates that the dust plume originates from the Al-Nefud desert in the Arabian Peninsula as well as regions in South Mesopotamia at around 06:00 UTC, 21 April. The subsequent transport of this dust plume toward Tehran is also shown in the “Supplement Movie”. The ECMWF reanalysis of surface wind speed and direction at 06:00 UTC, 21 April 2015 (Fig. 8a) reveals that a very strong surface wind which its speed reaches almost to 15 m/s, blows over Al-Nefud desert and south of Iraq (dashed oval in Fig. 8a). The wind speed exceeds the minimum value of threshold friction velocity and has the potential to activate dust sources in this region^55^. The daily Aqua MODIS deep blue AODs over the region on 21 April 2015 are illustrated in Fig. 8b. Areas with AOD$~>1$ are mostly located in the region between the north of Saudi Arabia and the south of Iraq which is consistent with the dust source activation in these regions (Fig. 8a). Depolarization ratios from nighttime CALIOP measurements on 21 April 2015 are shown in Fig. 8c. Figure 8b,c reveal that a lofted dust layer (up to 5 km) has been formed over the north of Saudi Arabia and the south of Iraq ($\sim 28^{\circ }$ N–$34^{\circ }$ N). CALIOP recordings are in good consistency with the depolarization ratio measured by our lidar station ($\delta _{\text {p}}\sim 0.34$), and it also corroborates the large AOD values observed by MODIS over Iraq and Saudi Arabia (Fig. 8b). The ECMWF reanalysis of the atmospheric vertical velocity at 06:00 UTC, 21 April 2015 on 850 hPa (color) and surface wind speed (arrows) are depicted in Fig. 8d. The atmospheric vertical velocity over the expected source (dashed oval in Fig. 8d) is mainly negative, which is an indicator of an upward movement of air parcels. The wind profile at 850 hPa (Fig. 8e) depicts a very strong south-westerly wind over the source region and clearly confirms that the lofted dust plume subsequently is transferred to the west and central part of Iran. The 72 HYSPLIT backward trajectories started from the lidar station on 22 April 2015 at altitudes of 2500 m agl and 2200 m agl are returning back to the Arabian Peninsula and south of the Mesopotamia respectively (Fig. 8f). Daily values of deep blue AODs which are captured by Terra MODIS on 22 April 2015 (Fig. 8g) clearly indicate the eastward transport of the dust plume towards the Iran Plateau. Due to the movement of this dust plume, AODs over the west and central parts of Iran reach to values even higher than 1.0.

To conclude, the MSG-SEVIRI RGB dust composite, ECMWF reanalysis, MODIS Deep blue AOD, CALIOP depolarization ratio measurements, HYSPLIT backward trajectories, and our lidar measurements have good consistency with each other and confirms that some dust plumes are originated from the regions in the north of the Arabian Peninsula and South Mesopotamia and impacted Tehran.

### Boundary layer height evolution

To have a better understanding of atmospheric pollution episodes in Tehran, the planetary boundary layer height (PBLH) is retrieved from lidar recordings^38,39^. The PBLH algorithm, which is explained in “Planetary boundary layer height calculation”, is carried out on all lidar recordings and the top height of the polluted boundary layer has extracted for each day during the lidar measurement campaign (Nov. 2014 until Jan. 2016).

**Figure 9:**
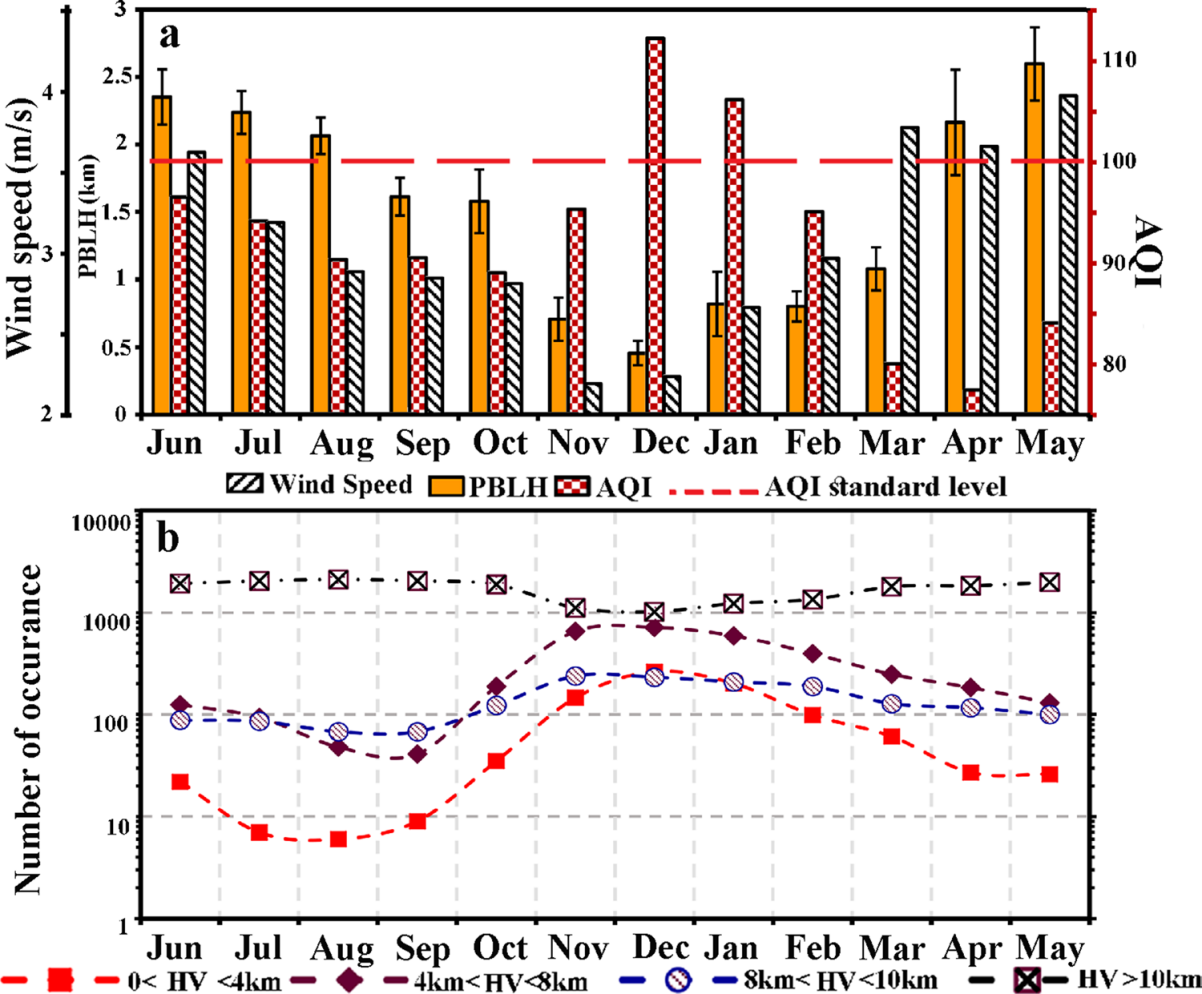
(**a**) Monthly average of retrieved PBLH during lidar campaign, air quality index (AQI) and surface wind speed over the period between 2010 and 2018. (**b**) Number of occurrences of the 3-h recorded HV per month by values appeared in the legend of the figure, during years 2010 to 2018.

Figure 9a illustrates monthly averaged values of the PBLH which are retrieved from the lidar recordings, monthly averages of air quality index (AQI), and surface wind speed. The AQI is calculated based on the concentrations of different air pollutants (carbon oxides, Nitrogen oxides and PMs)^56,57^. The AQI is averaged over recordings performed on all active AQCC stations in Tehran (Fig. 1a) and surface wind speed data are recorded at Mehrabad station during the years 2010 to 2018. The 3-h HV recordings at the Mehrabad station from 2010 to 2018 are also analyzed and the number of occurrence of events with different HV is shown in Fig. 9b. Events with decreasing HV due to precipitation, are excluded from the data set.

During cold months of the year (Nov.–Feb.), the PBLH remains below 1 km and even drop to $\sim$500 m gl in December. The monthly average of AQI also raises from Nov. to Feb. This index exceeds the WHO standard level ($\hbox {AQI}> 100$ means unhealthy air for sensitive group^2^) during January and has its peaks in December. All these developments coincide with a decrease of the monthly average of surface wind speed to $\sim$ 2 m/s from Nov. to Dec. On the other hand, during other months with better air quality conditions in which the AQI is below the standard level, the PBLH and the average of surface wind speed increases. Therefore, meteorological factors such as temperature inversion (low PBLH) and stable atmosphere (low wind speed) during cold seasons lead to the accumulation of particulate matters in the planetary boundary layer and eventually cause a reduction of the HV. Figure 9b shows number of days per months, for the 9 years of recordings (2010–2018), when the HV was at four different ranges: 10 $\hbox {km}< \hbox {HV}, \hbox {8km}<\hbox {HV}<10\, \hbox {km}, 4\, \hbox {km}< \hbox {HV} <8\, \hbox {km}$, and $\hbox {HV} < 4\, \hbox {km}$. One can find from Fig. 9b that lower values of the HV mostly are happening during November–January. It is noticeable that during winter times anthropogenic activities are responsible for production of more than 70% of fine particulate^6^. These reflect the importance of urban-industrial aerosols in atmospheric pollution of Tehran especially during cold months of the year.

### Source apportionment

**Figure 10:**
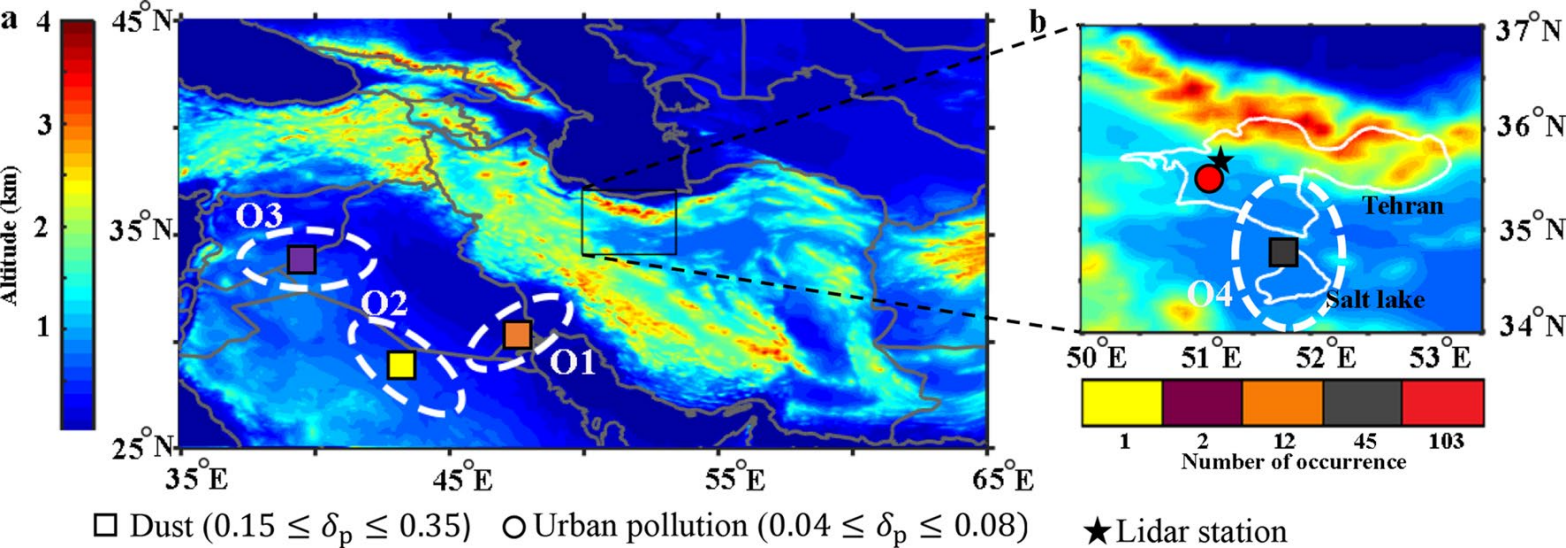
Number of occurrences (horizontal color-bar), approximate origins (white ovals) of the observed dust (squares) and urban pollution (circles) events over Tehran during Nov. 2014 to Jan. 2016 based on lidar measurements, MODIS AOD’s and HYSPLIT back trajectories, over the orography map of (**a**) Iran and its neighboring countries, (**b**) Tehran province and its neighboring regions.

Figure 10 shows distribution of atmospheric aerosol sources that had some impacts over Tehran during the measurement campaign. These sources are specified based on HYSPLIT backward trajectories, together with aerosol type categorization that are retrieved from lidar depolarization ratio recordings. Dashed ovals determine approximate regions of expected sources, and the surface topography is shown in a rainbow color tone in Fig. 10. Borders of the greater Tehran province and the salt lake located on South Tehran are shown in Fig. 10b.

The lidar was in operation for 357 days during the measurement campaign. In this period, there are 163 days in which either ${{\text PM}_{2.5}}$ or ${{\text PM}_{10}}$ concentrations are increased above the WHO standard levels^2^. These events are classified as dust (square) or urban pollution (circle) based on the retrieved depolarization ratios for atmospheric particles. Number of events where the atmosphere was polluted with aerosols from different origins are shown in five different colors (Fig. 10). Urban pollution ($0.04 \le \delta _{\text {p}} \le 0.08$) is the most recorded case (103 cases). It is expected that such particles come from local source in the city. In total, 45 dust events are originated from regions in the south of Tehran (O4 in Fig. 10b). Our recordings show that the Qom-Salt-Lake, in the south of Tehran, had considerable impacts at least on 4 cases of dust events during the measurement campaign. The depolarization ratio for dust cases which are originated from local sources ($0.15<\delta _{\text {p}}<0.2$) is lower compared to dust events from external sources ($\delta _{\text {p}}\sim 0.31$). During the measurement campaign, the highest recorded depolarization ratio was for a Haboob dust storm outbreaks ($\delta _{\text {p}}\sim 0.35$) which its details has not presented in this paper. The number of dust storms which are originated from the south of Mesopotamia (O1) reaches to 12 events. In total, the Arabian Peninsula (O2), Syria and Iraq (O3) are recognized as sources of three dust storms during the measurement campaign.

## Summary and conclusion

For the first time, polarization lidar observations are performed to monitor the highly polluted atmosphere above Tehran, Iran. Lidar measurements are carried on during November 2014 to January 2016. The ultimate outcomes of this study are to determine types, sources, and annual cycles of particulate matters in the atmosphere above Tehran.

To explain the atmospheric pollution condition in Tehran, three cases are discussed as illustrative examples in this article. The first case presents a high load of aerosols in the planetary boundary layer of Tehran’s atmosphere, while the particle depolarization ratio of the polluted layer is predominantly low ($\delta _{\text {p}}\sim 0.07$). The lidar derived mass concentration of the atmospheric aerosols at the vicinity of the surface is retrieved as 130 $\upmu {\text{g/m}}^{3}$, which is in an acceptable agreement with ${{\text PM}_{10}}\sim 150$ $\upmu {\text{g/m}}^{3}$ reported by the AQCC. These kinds of pollution events usually are happening during the cold months of the year in Tehran (Nov.–Feb.). During these kinds of pollution episodes, the HV drops to $<4$ km and even $<2$ km in Tehran. Case 2 shows how mineral particles ($\delta _{\text {p}}\sim 0.17$) from salt lakes in the south of Tehran may impact the atmosphere of the city. The long-range dust transport from Mesopotamia and the Arabian Peninsula toward Tehran is described in the last case. The particle depolarization ratio for such a dust storm is recorded in the range of $0.29\le \delta _{\text {p}}\le 0.34$. During this dust outbreaks, the surface wind speed is increased to 8–10 m/s, and the HV dropped to $\le 4$ km in Tehran.

Monthly evolution of the PBLH over Tehran shows a seasonal dependence. From November to February, while atmospheric temperature inversion frequently happens, the PBLH mostly remains below 1 km and even drops to $\sim 500$ m agl in December (Fig. 9a). Investigation of monthly variations of the PBLH, the AQI and surface wind speed reveals that during air pollution episodes, atmospheric temperature inversion and low wind speed cause that atmosphere becomes completely stable and polluted layers stay close to the ground level and eventually lead to high PM concentrations (Fig. 9a). In other words, seasonal variations of meteorological factors besides anthropogenic activities have considerable impacts on urban pollution in Tehran.

During the measurement campaign, there are 103 cases in which pollutant particles are originated from anthropogenic activities. This work also emphasizes that the dry regions in the south of Tehran province, including the Qom dry lake, as origins of 45 recorded dust events, are quite influential on aerosol loading into the atmosphere above Tehran (Fig. 10b). It is interesting that regions in the south of the Mesopotamia (Fig. 10, O2) have considerable impacts on dust contents of the Tehran’s atmosphere. Twelve dust events have been recorded with origins from these area during the measurement campaign. From the recordings one may mention two dust events that were originated from the Syrian desert and an intense dust outbreaks with origins in the Al-Nefud desert in the north of the Arabian Peninsula.

## Supplementary information

Supplementary Information.

Supplementary Video.
