# Supplementary material for: Monitoring atmospheric particulate matters using vertically resolved measurements of a polarization lidar, in-situ recordings and satellite data over Tehran, Iran
Source: Sci Rep. 2020 Nov 18;10:20052. doi: 10.1038/s41598-020-76947-w (PMC7676275; doi:10.1038/s41598-020-76947-w)
Supplement: Supplementary file 1 — Supplementary Information. [file 41598_2020_76947_MOESM1_ESM.pdf]

Title of the manuscript: Monitoring Atmospheric Particulate Matters Using Vertically Resolved Measurements of a Polarization Lidar, In-situ Recordings and Satellite Data Over Tehran, Iran

List of authors: Hossein Panahifar, Ruhollah Moradhaseli, Hamid Reza Khalesifard

Caption for the supplementary movie: A dust plume is started to form at ~06:00 UTC on 21 April 2015 from the Al-Nefud desert on the north of the Arabian Peninsula. The plume is transported toward the northeast and reaches to Tehran on 22 April 2015. This is in good agreement with our lidar measurements. The Dust product is an RGB (Red, Green, Blue) composite based upon SEVIRI IR8.7, IR10.8 and IR12.0 channels from the Meteosat Second Generation satellite.
